# A Survey on Congestion Control for RPL-Based Wireless Sensor Networks

**DOI:** 10.3390/s19112567

**Published:** 2019-06-05

**Authors:** Chansook Lim

**Affiliations:** Department of Software & Communications Engineering, Hongik University, Sejong 30016, Korea; chansooklim@hongik.ac.kr; Tel.: +82-44-860-2549

**Keywords:** RPL, congestion control, load balancing, IoT, WSN

## Abstract

RPL (IPv6 routing protocol for low power and lossy networks) proposed by the IETF (Internet Engineering Task Force) ROLL (routing over low-power and lossy networks) working group is a de facto standard routing protocol for IoT environments. Since the standardization was proposed, RPL has been extensively improved for diverse application scenarios and environments. Congestion control is one of the most important reasons why RPL has been improved. In an LLN (low power and lossy network), congestion may even lead to network lifetime reduction. In resource-constrained networks where end-to-end congestion control is not feasible, RPL should play a more crucial role in congestion control. In this survey, we review the RPL schemes proposed for congestion control and load-balancing and discuss future research directions.

## 1. Introduction

IoT (Internet of Things) is one of the key technologies enabling the next industrial revolution. As the IoT industry evolves rapidly, IoT standardization has been done by more than 100 working groups over the world. The IETF (Internet Engineering Task Force) also has many working groups (WGs) focusing on IoT standardization for resource-constrained devices and networks. For instance, the 6lo (IPv6 over networks of resource-constrained Nodes) WG defines methods for adapting IPv6 to constrained node networks. The ROLL (routing over low-power and lossy networks) WG focuses on routing issues for resource-constrained devices. The CoRE (constrained restful environments) WG was chartered to provide a framework for resource-oriented applications intended to run on constrained IP networks. The ACE WG focuses on authentication and authorization mechanisms in IoT and the 6TiSCH (IPv6 over the TSCH mode of IEEE 802.15.4e) WG issues on adding the TSCH (time-slotted channel hopping) mode to IEEE 802.15.4e. In this survey, we are focused on RPL (IPv6 routing protocol for low power and lossy networks) [1] standardized by the ROLL WG.

In a low-power and lossy network (LLN), routers typically operate with constraints on processing power, memory, and energy. Therefore, LLNs are characterized by high loss rates, low data rates, and instability. RPL was designed with the objective to meet routing requirements that the IETF ROLL working group defined for four LLN application domains: urban LLNs, industrial LLNs, home automation LLNs, and building automation LLNs [2,3,4,5], but RPL aims to be useful in a wide range of LLN application domains. For wide applicability, RPL separates packet processing and forwarding from routing optimization objectives such as minimizing energy, minimizing latency, and satisfying constraints.

In compliance with the standard, RPL has been enhanced, optimized, and implemented to meet the requirements for the target applications. Depending on the application scenario, the issues to be resolved are different. One of the common issues is congestion. In a large LLN, even low-rate traffic generated by each node can cause congestion at areas where the traffic is concentrated.

Congestion causes packet losses and delay in the first place. Packet losses in LLNs also may affect reliability and robustness of the applications if end-to-end congestion control and packet recovery is not ensured by the upper layer protocol due to resource limitations. In addition, congestion wastes energy and shortens the network lifetime. Load-balancing is a relevant issue to the network lifetime and congestion. Nonuniform energy depletion due to unbalanced workload distribution will shorten the overall network lifetime. Load-balancing can be utilized to alleviate congestion.

Recently, several insightful surveys on RPL have been proposed. Iova et al. surveyed the current status of RPL based on the original requirement defined by the IETF and discussed new requirements taking the current IoT trends into account [6]. Kim et al. provided a pioneering survey which reviews the history of research efforts in RPL from a variety of viewpoints [7]. They present a topic-oriented survey for each of 13 subtopics related to RPL, including load balancing. Kamgueu et al. performed a survey on RPL enhancements focusing on topology, security, and mobility [8]. Ghaleb et al. conducted another comprehensive survey [9] covering background knowledge on LLNs to RPL enhancements. Since [9] reviews RPL enhancements and extensions related to OFs, downward routing, and route maintenance, it includes congestion control and load balancing within its scope, and gives insights on the solutions to congestion via OF enhancements. However, Ghaleb et al. [9] classifies OF enhancements by routing metrics and multipath routing, not by the main purposes such as congestion control, which means that the readers interested in a specific purpose of RPL enhancement need to make additional efforts to understand the relevant literature. Compared to prior surveys, especially the two surveys [7,9] which are of vast scope, this survey is narrow in scope, with the focus on congestion control and load balancing. However, this survey reviews several publications which are not reviewed in [7] or [9] and aims to analyze research efforts in the literature of congestion control in more detail.

The rest of the paper is organized as follows. Section 2 provides an overview of RPL. Section 3 discusses classification and Section 4 summarizes each of the congestion control schemes for RPL. Section 5 discusses research challenges. In Section 6, we draw conclusions.

## 2. Overview of RPL

RPL [1] is a proactive, distance-vector routing protocol for low-power and lossy networks (LLNs) which are characterized by high loss rates, low data rates, and instability. In LLNs, routers typically operate with constraints of processing power, memory, and energy.

RPL supports multipoint-to-point, point-to-multipoint, and point-to-point traffic flows which correspond to upward, downward, and any-to-any traffic patterns, respectively. Typical application scenarios such as monitoring in LLNs require multipoint-to-point traffic flows from the sensing devices to the central control point. Applications such as actuation and selective sensor queries generate point-to-multipoint traffic flows which need downward routes. Since RPL is based on LLNs comprising IPv6 addressable nodes, we can envision diverse point-to-point IoT applications where a device interacts with another device within an LLN.

### 2.1. DODAG Construction for Multipoint-to-Point Traffic Flows

**Figure 1:**
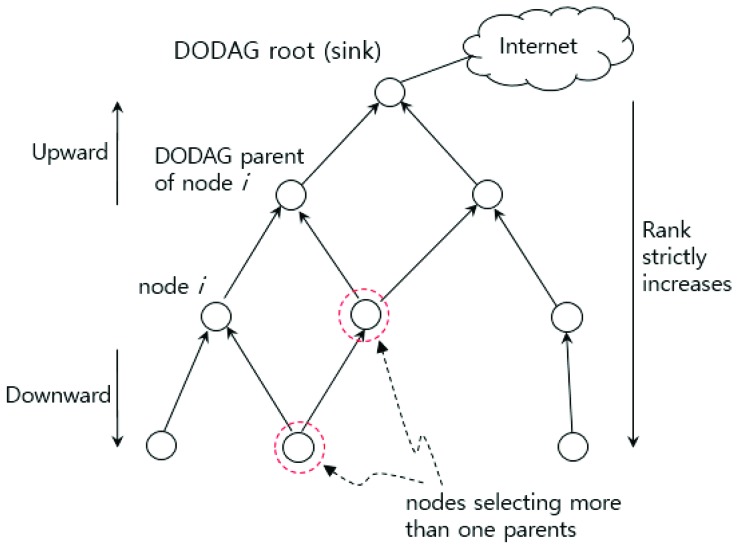
An illustration of upward routing in RPL.

RPL constructs a topology as a directed acyclic graph (DAG) which has one or more roots. In RPL, a DAG root is a node within the DAG that has no outgoing edge, as shown in Figure 1. The DAG can be partitioned into one or more destination-oriented DAGs (DODAGs), with one DODAG per sink. A DODAG root may act as a border router for a DODAG, may aggregate routes in the DODAG, and may redistribute DODAG routes into other routing protocols. Since RPL was designed for lossy network environments, each node in a DODAG maintains a set of candidate parents for fault tolerance purposes. From the set, each node selects one or multiple preferred parents as the primary relay node(s) for packet delivery.

Each node in the DODAG is assigned a rank. The rank of a node indicates the node’s individual position relative to other nodes with respect to a DODAG root. The rank strictly increases in the Down direction, (i.e., from DODAG roots toward the leaf nodes) and decreases in the Up direction. The rank is used to avoid and detect loops created due to topology changes. The rank is not a path cost, but can be derived from path metrics based on the DAG’s objective function (OF).

DODAG construction begins with the root node broadcasting a DAG information object (DIO) message, which includes RPLInstanceID, the DODAG identifier, a monotonically increased version number, rank, and other fields. The nodes closest to the root will first hear this message, and decide whether to join this DODAG. If they want to join, they compute their own rank values independently, and transmit DIO messages containing their own rank values to their neighbors. If a node learns that it has already received a copy of the DIO message, it remains silent. Through DIO messages, RPL nodes select and maintain upward routes toward the DODAG root according to an objective function defining how to select and optimize routes. Note that a DODAG is constructed to optimize performance for multipoint-to-point traffic.

A node transmits a DIO message using a Trickle timer (RFC6206) [10]. The Trickle algorithm quickly propagates updated information when inconsistency is detected among nodes, whereas it slows the sending rate down exponentially when nodes agree and therefore information is not changed. Through this adaptive transmission interval adjustment and the suppression mechanism, the Trickle algorithm achieves scalability and energy efficiency.

### 2.2. Route Construction for Point-to-Multipoint and Point-to-Point Traffic Flows

To construct and maintain downward routes from a DODAG root toward leaves, RPL nodes send destination advertisement object (DAO) messages upward. Each node sends a DAO message to one of its DAO parents which are in the node’s DODAG parent set. An RPL instance may choose between two modes for maintaining downward routes. One is “storing” mode where a node stores a downward routing table for its sub-DODAG. The other mode is “nonstoring” mode where nodes do not store downward routing tables and packets are routed with source routes constructed by the DODAG root. Hence, in nonstoring mode, the DODAG root is expected to store source routing information learned from DAO messages.

In RPL, point-to-point traffic uses both upward routes and downward routes. A point-to-point packet flows towards a DODAG root until it reaches an ancestor that has a known route to the destination. If nodes cannot store routes, the common ancestor may be the DODAG root. The main drawback of point-to-point routing specified by the RPL standard is that the routes from the source to the destination can be suboptimal and congestion can take place near the root. Also, in contrast with the proactive nature of RPL, applications such as home automation and building automation require a device to communicate with another device on demand. To address the drawbacks, an extension of RPL, P2P-RPL [11,12], has been proposed as an experimental standard. P2P-RPL provides a reactive mechanism. When a node (source) needs to communicate with another node (destination), the source initiates the route discovery process by forming a temporary DAG rooted at itself. While this DAG exists, the routes are discovered and installed. The installed routes remain for the specified lifetime even though the temporary DAG no longer exists. The DIOs used to form the temporary DAG are identified using the MOP (mode of operation) indicating P2P route discovery mode. A P2P mode DIO carries the destination address, the nature of the route to be found (i.e., hop-by-hop or source routes), etc. Propagation of P2P mode DIO messages can be limited by using aggregated routing metrics and constraints carried in the DIO messages.

### 2.3. Objective Function and Routing Metrics in RPL

RPL supports both static and dynamic metrics. RPL also supports constraint-based routing. If a link or a node does not satisfy a required constraint, it is pruned from the candidate neighbor set. RFC6551 [13] specifies a set of routing metrics and constraints suitable for RPL.

An objective function (OF) defines how a node computes the rank and selects its preferred parent. The rank is computed using routing metrics, optimization objectives, and related functions. Each RPL instance is associated with an OF specialized for it. The RPL standard described in RFC 6550 [1] does not suggest any specific OF. Instead, there are separate specifications defining objective functions. RFC 6552 [14] defines the Objective Function Zero (OF0) designed as a default OF that allows interoperation between implementations in a wide spectrum of use cases. OF0 is designed to find the nearest DODAG root that provides connectivity, though it does not guarantee path optimization with respect to a specific metric. The OF0 selects a preferred parent and a backup feasible successor if one is available. The minimum rank with hysteresis objective function (MRHOF) described in RFC 6719 [15] selects routes that minimize a metric, while using hysteresis to reduce churn in response to small metric changes. MRHOF works with additive metrics along a route. Typical examples of metrics for MRHOF include latency metric and expected transmission count (ETX) metric, i.e., the expected number of transmissions required to send a packet over a link, including retransmissions. For example, if MRHOF is used with a latency metric, RPL tries to find a minimum latency path from the node to a DODAG root. In the absence of a metric in a DAG metric container, MRHOF is supposed to use ETX metric by default to find a minimum ETX path from the node to the root. When finding a path with the minimum rank, MRHOF switches to that minimum rank path only if it is shorter than the current path by at least a given threshold.

### 2.4. Supporting Multiple RPL Instances

A network may run multiple RPL instances concurrently. Each instance is logically independent in that each instance may serve different constraints or performance criteria. A node may belong to multiple RPL instances. A network may consist of one or several DODAGs which together form an RPL instance identified by an RPLInstanceID.

## 3. Classification of Schemes for Congestion Control and Load Balancing in RPL

Schemes for congestion control and load balancing in RPL can be classified based on the common criteria used in the literature surveys of wireless sensor networks as follows.

- Congestion detection
- Congestion notification
- Congestion mitigation

We can classify the schemes based on some major design factors specific to RPL, as follows.

- Traffic pattern (upward, downward, and any-to-any)
- Routing metrics
- Adopting cross-layer approaches
- Utilizing path diversity

**Congestion detection**. Most schemes surveyed try to forward packets or distribute traffic through less congested routes by using routing metrics indicating degree of congestion. Some of them explicitly detect congestion using detection metrics as shown Table 1. We note that all these schemes, except M-RPL, are single-path mechanisms (M-RPL performs temporary multipath routing on detecting congestion).

**Congestion notification.** In all the schemes in Table 1, when detecting congestion, a node sends its children DIO messages containing congestion information. In particular, GTCC [16], CoAR [21], and M-RPL [22] explicitly set the congestion notification (CN) bit set in the header.

**Congestion mitigation.** According to recent surveys on congestion control for WSN [23,24,25], the approaches to congestion mitigation are classified into at least two groups involving traffic control and resource control.

- Resource control: Resource control is related to provisioning network resources to mitigate congestion. Alternative path selection schemes, multipath schemes, power control, and so on, belong in this category. Most RPL schemes proposed for congestion control adopt resource control approaches including route change, multipath forwarding, power control, and multisink.
- Resource control combined with traffic control: A traffic control approach attempts to mitigate congestion by adjusting the sending rate. Since the primary role of a network layer protocol is to find a good route, RPL schemes are rarely related to adaptation of the sending rate. Nevertheless, some schemes, such as GTCC [16] and OHCA [20], adopt both resource control and traffic control approaches.

**Traffic patterns.** Naturally, multipoint-to-point traffic is the first target which most studies are focused on. Yet, there are some studies that pay attention to congestion in an environment with point-to-multipoint traffic. As mentioned in Section 2, downward routes for point-to-multipoint traffic are established using DAO packets. Since DAO packets are transmitted upward along the DODAG, they may cause congestion. For instance, if many DAO packets are generated due to global repair, but buffer space is not sufficient, DAO packets as well as data packets may be lost. Tripathi et al. deal with this congestion problem due to DAO packets [26].

As described in Section 2, routes for point-to-point traffic can be established in one of two ways: upward routing combined with downward routing as specified in RPL standard or reactive routing initiated by the source as specified in P2P-RPL. The former is known to have several drawbacks, including congestion near the sink. The latter has been proposed to address the shortcomings of the former, but needs to be examined further.

**Routing metrics.** Routing in LLNs needs to support both link and node metrics. Table 2 shows the component metrics that the schemes reviewed in this survey use for parent selection or load distribution. The most frequently used metrics is ETX, which indicates link quality, reliability, and link-contention-based congestion. Also, as expected, many schemes monitor the degree of queue utilization to handle buffer-based congestion. Some opportunistic routing schemes use EDC which is an adaptation of ETX considering the number of duty cycled wakeups [27]. Some schemes use latency or data rates as a potential congestion indicator. It is worth noting that many schemes use link layer or physical layer metrics such as LQI, RSSI, channel capacity, busy channel probability, superframe distance, and residual energy.

There are several approaches to combining more than one routing metric in RPL. In [9], Ghaleb et al. categorize OF enhancements based on metric composition into lexical, additive, hybrid, fuzzy-logic based composition, etc. In lexical composition, a preferred parent is selected based on the primary metric and for tie-breaking, other metrics are used. Additive composition adds the weighted metric values to produce a new composite metric to be used for parent selection. Hybrid composition uses both lexical and additive techniques. For the methods of metric composition, we refer the readers to [9].

Approaches requiring exchange of a lot of metric information between nodes should consider a possibility of fragmentation pointed out in [9,42]. Since the maximum frame size at the 802.15.4 MAC layer is 102 octets, carrying a lot of information in a control message may cause the fragmentation problem, which may be exacerbated in multipath routing.

**Adopting cross-layer design approaches.** Since the definition of cross-layer design is not clear-cut, we adopt the definition and categorization proposed in [43] for our categorization. Cross-layer design is defined as violation of a layered architecture by allowing direct communication between protocols at nonadjacent layers or sharing variables between layers. If we categorize the cross-layer based schemes reviewed in this survey according to the basic ways of violating the layered architecture as presented in [43], most of them can be categorized into the group violating the layered architecture by “creation of new interfaces”. This category is further divided into three subcategories depending on the direction of information flow along the new interfaces: upward, downward, and back and forth. For instance, since GTCC [16] uses LQI from the physical layer, the direction of information flow is ‘upward’ (from a lower layer to a higher layer). Table 3 summarizes the cross-layer design proposals reviewed in this survey, indicating the direction of information flow.

**Utilizing path diversity.** RPL provides a framework where a node can exploit path diversity by maintaining multiple parents. An obvious way to fully exploit path diversity for congestion mitigation is to forward packets via multiple parents on a per-packet basis. Section 4.2 presents many schemes adopting this approach.

## 4. Congestion Control Schemes for RPL

In this section, we summarize each of the schemes reviewed. Although it is feasible to categorize them by any of the classification criteria described in the previous section, we divide the schemes in two categories based on whether they forward packets via a single parent or multiple parents.

### 4.1. Schemes Forwarding Packets via a Single Parent

We first summarize the congestion control schemes forwarding packets via a single parent. These RPL schemes use a parent node to forward packets towards the root until a new parent node needs to be chosen. Here, we also include the schemes which do not explicitly specify whether or not they forward packets through multiple parents. Table 4 at the end of this section summarizes the main feature, routing metrics, and evaluation tools and metrics for each scheme.

#### 4.1.1. GTCC

Sheu et al. propose a game theory-based congestion control protocol (GTCC) to mitigate the effect of congestion by changing the parent [16]. GTCC detects congestion by monitoring the net packet flow rate which is the packet generation rate subtracted by the packet service rate. If the net packet flow rate is positive, the probability of congestion is deemed high. Once congestion is detected, a parent node sends its children a DIO packet with the CN (congestion notification) bit set. The DIO packet also carries the parent information, children list, corresponding transmission rate, and LQI. Upon receiving DIO packets with the CN bit set from its neighbors, nodes in the congested area carry out the parent-change procedure based on potential game theory.

The rank of a node is calculated using the rank of its parent and LQI. Each node tries to select a parent which minimizes its rank. Selection of the same parent by too many nodes may lead to congestion. In GTCC, this problem is modeled as a potential game called the parent-selection game. In a potential game, all nodes (players) can reach a Nash equilibrium if only one node (player) is allowed to change its parent at a time. A player set consists of children nodes which receive the same DIO packet with a CN bit set from the same parent node. GTCC allows only one node to change its parent at a time by using a random timer. The incentive for each node to select its parent is expressed by the potential function which is a single global function. As input, the potential function takes the rank of a parent candidate, LQI and the transmission rates between the parent candidate and its children, and the number of children of the parent candidate. In each round, each node finds a new parent which can decrease the value of the potential function. To avoid the ping-pong effect of changing the parent frequently, GTCC adds a hysteresis loop in calculating the rank using LQI. If congestion is not mitigated after the parent change procedure, GTCC performs the rate adaptation procedure to reduce the sending rates of the sources.

The authors compared performance between GTCC and two ContikiRPL protocols with OF0 and OF-ETX using Cooja simulator. Simulation results show that GTCC achieved higher throughput and a lower packet loss rate than the other two ContikiRPL protocols as the transmission rate grew.

A drawback of this scheme is that in order to calculate the potential function, each node needs the information on the transmission rates between parent candidates and their children nodes. In a dense network, the amount of the information carried in a DIO message notifying congestion can increase along with the number of children.

There are other studies which adopt a game theoretic approach to the congestion problem in RPL-based networks. For instance, GTCCF [44] proposed by Al-Kashoash et al. formulated the congestion problem in a 6LoWPAN network as a noncooperative game problem. However, since GTCCF is a framework for rate adaptation on a fixed DODAG topology, we omit the details.

#### 4.1.2. QU-RPL

In [17], Kim et al. propose a queue utilization based RPL (QU-RPL) to tackle the load balancing and congestion problem. In their preliminary study, they performed experiments using TinyRPL with OF0 in a real testbed. TinyRPL with OF0 uses a hop count and ETX as routing metrics. They observed that ETX may not be very high even for heavy traffic load if the link capacity is greater than the buffer capacity. They made a modification on TinyRPL so that queue utilization is considered for parent selection.

To detect congestion, QU-RPL uses queue utilization (QU) factor. For each node *k*, QU factor Q(*k*) is defined as follows.

(1) $$Q\left(k\right)=\frac{\#\;\text{of packets in queue of node}\;k}{\text{the total queue size of node}\;k}$$

When parent node *p_k_* is severely congested, Q(*p_k_*) tends to be significantly greater than Q(*k*). In this case, it is desirable for node *k* not to be selected as a parent by its neighbors, because node *k* must forward all packets received from children nodes to parent *p_k_*. To this end, Q(*k*) is adjusted after selecting a parent *p_k_* as follows.

(2) $$Q\left(k\right)=\max\left\{Q\left(p_{k}\right)-\lambda ,\;Q\left(k\right)\right\}$$

where λ is a small positive QU reduction factor which makes Q(*k*) < Q(*p_k_*). For example, if *λ* is 0.25, a congested node can trigger QU adjustment for up to three-hop children nodes. The routing metric for a parent candidate *p_k_* is defined as follows.

(3) $$R_{QU}\left(p_{k}\right)=h\left(p_{k}\right)+1+ETX\left(k,p_{k}\right)+\alpha *Q\left(p_{k}\right)$$

where *h*(*p_k_*) is the hop count between node *p_k_* and the LBR (LLN border router), *ETX*(*k*, *p_k_*) is ETX for the link between node *k* and node *p_k_*, and α is a coefficient which controls the weight for QU factor. A parent candidate with the minimum routing metric is selected as the best alternative parent.

QU-RPL allows a node to consider a parent change when both the stability condition and congestion condition are satisfied. The stability condition inherited from TinyRPL allows a node to change its parent only when the routing metric of its best parent candidate exceeds that of the current parent by a stability bound. The congestion condition is met when the congestion indicator exceeds the given threshold. As the congestion indicator, each node uses the maximum value among the QU factors of all the parent candidates that the node had in the recent past. If both conditions are satisfied, a parent change is made probabilistically in order to avoid the herding effect.

The authors verified the performance of QU-RPL through experiments on a real testbed where 30 LLN endpoints and one LBR communicate with each other through IEEE 802.15.4 links. They used the default CSMA of TinyOS, and did not use a duty cycling mechanism. Experimental results show that compared to TinyRPL, QU-RPL significantly reduces the average queue loss ratio, thereby enhancing the PRR significantly for most of the nodes.

#### 4.1.3. Residual Energy-Aware Congestion Control

Ullah et al. propose an energy- and congestion-aware routing protocol for smart grid AMI networks [18]. This scheme uses a new metric called ECRM which considers the residual energy as well as ETX and the queue utilization factor of a neighbor node. Each node calculates its own rank by adding a constant MinHopRankIncrease to its parent’s rank. Each node *k* calculates a metric $R_{Pro}\left(p_{k}\right)$ for its parent $p_{k}$ as follows.

(4) $$R_{pro}\left(p_{k}\right)=Rank\left(p_{k}\right)+ETX\left(k,p_{k}\right)+\alpha Q\left(p_{k}\right)+\beta E_{res}\left(p_{k}\right)$$

where $Q\left(p_{k}\right)$ is the queue utilization factor, $E_{res}\left(p_{k}\right)$ the residual energy of $p_{k}$. The design parameters α and β are set to high values to avoid congested nodes and energy-constrained nodes, respectively. This scheme performs the parent change procedure when the residual energy is lower than a residual energy threshold or the queue utilization is higher than a predefined threshold.

To evaluate performance, the authors compared their scheme (using ECRM) to the energy-aware scheme (ELPS) proposed by Nassiri et al. [38] using Cooja simulator. They used the beacon-enabled 802.15.4 MAC protocol. Simulation results show that ECRM outperforms ELPS in terms of the average power consumption and packet delivery ratio.

#### 4.1.4. PC-RPL

Kim et al. proposed PC-RPL (power-controlled RPL) to address both the load imbalance and hidden terminal problem [19]. Their work was motivated by the observations in the preliminary experiments using RPL. As traffic load increased, most packet losses occurred at links rather than queues and the link losses were caused by packet collision due to the hidden terminal problem. Also, when packets were severely lost at queues, such losses occurred only at a few bottleneck nodes with large unbalanced subtrees. To address the problems, they designed a distributed and adaptive control mechanism where transmission power and routing topology are jointly controlled.

For parent selection, PC-RPL uses hop distance, ETX and RSSI. The most distinctive feature of PC-RPL is that it uses a reference RSSI value and adaptive RSSI thresholds. For each node *k*, the reference RSSI value for its neighbor $n_{k}$ is measured by making all nodes transmit DIO messages with the maximum transmission power without transmission power control. Node *k* includes its neighbor $n_{k}$ in its parent candidate set when

(5) $$RSSI_{ref}\left(k,\;n_{k}\right)\;>\;RSSI_{th}^{PS}\left(k\right)$$

where $RSSI_{ref}\left(k,\;n_{k}\right)$ and $RSSI_{th}^{PS}\left(k\right)$ are the reference RSSI value for $n_{k}$ and the ‘parent selection RSSI threshold’, respectively. If node *k* suffers from the hidden terminal problem, it increases $RSSI_{th}^{PS}\left(k\right)$ to select a ‘closer’ parent node. To improve load-balancing, node *k* adjusts the size of its subtree by enabling its potential children to select parents using adaptive RSSI thresholds. More specifically, a potential child node $n_{k}$ can add node *k* to its parent candidate set when

(6) $$RSSI_{ref}\left(n_{k},k\right)\;>\;RSSI_{th}^{CC}\left(k\right)$$

where $RSSI_{ref}\left(n_{k},k\right)$ is the reference RSSI value that $n_{k}$ has for node *k* and $RSSI_{th}^{CC}\left(k\right)$ is the ‘children control RSSI threshold’ that node *k* maintains and sends to $n_{k}$. When node *k* detects load imbalance indicated by frequent queue losses, it increases $RSSI_{th}^{CC}\left(k\right)$ to detach its children nodes. Upon increasing $RSSI_{th}^{CC}\left(k\right)$, node *k* propagates the increased threshold to its children by transmitting a DIO message. Consequently, a neighbor $n_{k}$ can be added to the parent candidate set of node *k*, when it satisfies the following condition.

(7) $$RSSI_{ref}\left(k,\;n_{k}\right)\;>\max\left\{RSSI_{th}^{PS}\left(k\right),\;RSSI_{th}^{CC}\left(n_{k}\right)\right\}$$

When a node *k* changes its parent to $p_{k}$, it tries to use ‘just enough’ transmission power to reach its parent $p_{k}$ while maintaining reliability by configuring its data packet transmission power to $p_{k}$, $P_{tx}^{data}\left(k\right)$ as follows.

(8) $$P_{tx}^{data}\left(k\right)=P_{tx}^{DIO}-\left(RSSI_{ref}\left(k,\;p_{k}\right)-RSSI_{th}^{def}\right)$$

where $P_{tx}^{DIO}$ and $RSSI_{th}^{def}$ are the DIO transmission power fixed to 0 dBm and the default RSSI threshold set to the clear channel assessment threshold (-77 dBm) used for CSMA, respectively.

The authors evaluated performance on a real testbed consisting of 49 nodes and one border router. Each node was a TelosB clone with IEEE 802.15.4 radio that employed TinyOS default CSMA. The duty cycling mechanism was disabled. PC-RPL was compared to the basic RPL and QU-RPL, in terms of PRR, hop count, end-to-end ETX, parent change frequency, routing overhead, and total transmission count. Experimental results show that PC-RPL was effective in addressing the hidden terminal problem which QU-RPL cannot alleviate. PC-RPL achieved a much higher PRR than QU_RPL. Also, the parent change frequency of PC-RPL was smaller than that of QU-RPL for all transmit power settings, while maintaining a high PRR.

#### 4.1.5. CA-OF

The congestion-aware objective function (CA-OF) [28] proposed by Al-Kashoash et al. also uses ETX and buffer occupancy both as routing metrics. The design of CA-OF is based on the assumption that ETX and buffer occupancy need to be weighed differently depending the degree of traffic load. The authors argue that ETX is a more effective metric for light traffic load because the majority of packets are lost at the wireless channel. On the other hand, buffer occupancy is a more important metric for heavy traffic because the majority of packets are lost due to buffer overflow.

CA-OF combines ETX and buffer occupancy (BO) using a simple weighted sum as follows:

(9) $$\mathrm{Combined}\_\mathrm{Metric}=w_{1}*\mathrm{ETX}+w_{2}*\mathrm{BO}$$

where the weights $w_{1}$ and $w_{2}$ represent the percentage of the free buffer space and the occupied buffer space, respectively. For example, $w_{1}$ is set to 100% if the buffer is empty. When the buffer is full, $w_{2}$ is set to 100%.

Performance was evaluated using Cooja simulator, and ContikiMAC RDC was used. The authors compared CA-OF to OF0, ETX-OF, and energy-OF. For two different network scenarios, CA-OF outperformed the other OFs in terms of packet losses, throughput, PDR, and energy consumption.

#### 4.1.6. OHCA

Al-Kashoash et al. proposed OHCA (optimization-based hybrid congestion alleviation) which combines a resource control strategy with a traffic control strategy [20]. If a parent node detects the arrival rate exceeding the service rate, it deems the congestion condition to be met and sends its children nodes a DIO message containing congestion information. On receiving a DIO message, a child node applies the resource control strategy to select a noncongested parent. If a noncongested parent node is not found, the traffic control strategy is applied.

For resource control, OHCA models parent selection as a multicriteria optimization problem and solves it by using a multiattribute decision making (MADM) technique called grey relational analysis (GRA). In OHCA, the objective function uses three routing metrics, buffer occupancy, ETX, and queuing delay. For each parent candidate, the GRA procedure calculates the rank considering different characteristics of the routing metrics. The main advantage of GRA is simplicity, which is crucial in resource-constrained networks.

If the resource control strategy fails (i.e., a noncongested parent node is not found), a less congested parent is selected from the candidate parents, and then the traffic control strategy is applied based on the node priorities, application priorities, and the congestion information received from the selected parent. In OHCA, a node’s sending rate adaptation is modeled as a constrained optimization problem which is solved by using the NUM (network utility maximization).

Performance was evaluated via Cooja simulation using CSMA/ContikiMAC(RDC)/802.15.4 at the data link layer. They compared OHCA to QU-RPL and DCCC6 (duty cycle-aware congestion control for 6LoWPAN) [45]. Like OHCA and QU-RPL, DCCC6 detects congestion using buffer occupancy, but uses a rate adaptation approach considering the presence of duty cycling. Simulation results showed that OHCA outperformed QU-RPL and DCCC6 in terms of throughput, fairness, end-to-end delay, energy consumption, and packet loss rate.

#### 4.1.7. CoAR

CoAR proposed by Bhandari et al. [21] is another congestion-aware RPL variant which considers ETX, queue utilization, and residual energy as routing metrics. In CoAR, a node is deemed congested when its buffer occupancy exceeds its threshold, which is adaptive according to the current buffer occupancy and the ratio of incoming traffic to outgoing traffic. To rank the candidate parents, CoAR adopts TOPSIS, which is a multicriteria decision-making approach. TOPSIS selects the best alternative based on the shortest distance from the positive ideal solution and the furthest distance from the negative ideal solution. For tiebreaking, CoAR uses NI, which indicates the ratio of the number of downstream nodes to the number of all neighbor nodes. By selecting a candidate parent with the smallest NI, CoAR tries to avoid using nodes with many potential children and few parents.

The authors compared performance of CoAR to that of the basic RPL and ECRM [18] via Cooja simulation. At the data link layer, CSMA/ContikiMAC(RDC)/802.15.4 was used. Simulation results show that CoAR outperformed the basic RPL and ECRM in terms of PRR (packet receiving ratio), end-to-end delay, packet loss ratio, throughput, and energy consumption.

#### 4.1.8. MAC-Aware Routing Metrics

Marco et al. propose two new metrics exploiting the information from the MAC layer in order to estimate reliability more accurately and to enhance network lifetime via load-balancing while satisfying reliability constraints [29,46]. One is R-metric which represents the end-to-end reliability between a node and the sink. R-metric extends ETX at the MAC layer considering packet losses due to MAC contention. On the basis that the reliability of each link depends on the busy channel probability, the bad channel probability (LQI), and the MAC parameters, R-metric is calculated based on the probability that a packet is successfully transmitted over each link on a path, within a maximum number of backoffs and retransmissions at the MAC layer. Node *i* selects a parent *j* satisfying

(10) $$\underset{j\in \Gamma_{i}}{\max}\;R_{i,j}\;\cdot R\left(j\right)$$

where $R_{i,j}$ is the reliability of link (*i*, *j*), *R*(*j*) the end-to-end reliability between node *j* and the root, and Γ*_i_* the set of candidate receivers, i.e., the nodes that can progress packets towards the root. R-metric calculation starts from the root, progressing from parents to children in the DODAG. An advantage of R-metric over ETX is faster estimation.

The other metric, Q-metric, is used for load balancing in the network. Node *i* selects the forwarding parent by solving the following optimization problem:

(11) $$\underset{j\in \Gamma_{i}}{\min}\;P_{t}Q_{j}+P_{r}\left(Q_{j}-\lambda_{j}\right)\;s.\;t.\;\;R_{i,j}\cdot R\left(j\right)\geq R_{min}$$

where $P_{t},\;P_{r},\;Q_{j},\;\lambda_{j},\;R_{min}$ are the transmission power consumption, the receiving power consumption, the traffic forwarded by node *j*, the traffic generated by node *j*, and the required reliability of the given application, respectively. Thus, node *i* needs to obtain information about its own traffic and parent candidates’ traffic through DIO messages.

Performance was evaluated via Cooja simulation for the network topologies with seven nodes and 18 nodes. The unslotted IEEE 802.15.4 MAC was used at the data link layer [29]. Simulation results show that R-metric and Q-metric achieve better end-to-end reliability than a back-pressure algorithm [47] which uses a weighted ETX cost including the queue differential between the transmitter and receiver. In terms of balanced energy consumption, Q-metric showed better performance than R-metric and the back-pressure scheme.

#### 4.1.9. Congestion Control using Multiple Sinks

In WSNs, hotspots tend to form near the sink. Since hotspot nodes may deplete energy fast, the network lifetime may be reduced. To alleviate this problem, Farooq et al. extend RPL to support multiple sinks in a network [30]. The routing table of each node contains the information about the discovered sinks: sink id, RPL instance, parent, rank, tie-breaking metric value, a flag that shows whether the node has joined the DODAG or not. Receiving a DIO message about an RPL instance of interest, a node joins the DODAG if the advertised DODAG is rooted at a new sink or better than the existing DODAG. A source node selects a sink with the minimum hop count before forwarding the packet.

The authors examine three objective functions OF1, OF2, and OF3. All of them use the hop count to the sink as the primary metric, but differ in the tie-break rules. OF1 simply selects one out of parent candidates at random without using any tie-break metric. OF2 selects a parent in a greedy manner using a tie-breaking metric such as available bandwidth, delay, ETX, or buffer occupancy at the MAC layer. For tie-breaker, OF2 and OF3 use similar metrics, but the metrics for tie-breaker in OF3 are end-to-end metrics between the node and the sink.

Performance was evaluated through Cooja simulation using a grid network topology with 75 nodes. At the MAC layer, IEEE 802.15.4 CSMA/CA was used, and the radio duty cycling mechanism was disabled. DIO messages were transmitted every second instead of using a Trickle timer. Simulation results show that OF2 performs better than OF1 and OF3 and that for a moderate rate of data generation, performance of all of the three OFs increases as the number of sinks increases.

### 4.2. Schemes Exploiting Path Diversity

Since one of the direct ways to eliminate hot spots in a network is to split traffic via multiple paths, it is natural that a lot of RPL schemes split traffic using multiple forwarders. In this section, we summarize the RPL schemes that try to alleviate congestion by forwarding packets through multiple parents on a per-packet basis. Although some of them aim at reducing the delay or extending the network lifetime rather than at directly mitigating congestion, they are expected to be effective in mitigating congestion because they try to prevent hot spot formation. In Table 5 at the end of this section, we compare the schemes in terms of main features, routing metrics, and performance evaluation tools and metrics.

#### 4.2.1. BRPL

To improve throughput and adaptability to time-varying traffic load and mobility, Tahir et al. proposed BRPL (backpressure RPL) [31] which switches smoothly and adaptively between RPL and backpressure routing according to the network conditions. BRPL exploits additional capacities of the suboptimal paths in the presence of heavy traffic load.

Since RPL allows multiple instances to run in a network and a DAG for each RPL instance consists of one or more DODAGs (and one or more roots), the authors define a topology of RPL using the concept of multitopology routing and formalize the RPL protocol using a multicommodity model where each DAG corresponds to a commodity.

BRPL constructs a DAG for upward routing by combining OF ranking with network congestion gradients reflected by differential queue gradients. For a DAG *m*, each node *x* computes a weight $w_{x,y}^{m}\left(t\right)$ for each neighbor *y* at each time slot *t* as follows.

(12) $$w_{x,y}^{m}\left(t\right)=\theta_{x}^{m}\left(t\right){\tilde{p}}_{x,y}^{m}\left(t\right)-\left(1-\theta_{x}^{m}\left(t\right)\right)∆Q_{x,y}^{m}\left(t\right)c_{x,y}\left(t\right)$$

where

(13) $${\tilde{p}}_{x,y}^{m}\left(t\right)=p_{x,y}^{m}\left(t\right)+Rank_{y}^{m}\left(t\right)$$

(14) $$∆Q_{x,y}^{m}\left(t\right)=Q_{x}^{m}\left(t\right)-Q_{y}^{m}\left(x,t\right)$$

and $Q_{x}^{m}\left(t\right),\;Q_{y}^{m}\left(x,t\right),\;p_{x,y}^{m}\left(t\right),\;c_{x,y}\left(t\right)$ denote the queue backlog of node x, the queue backlog for the node *y* recorded in *x*’s neighbor table, the penalty(cost) of using the link (*x, y*), and the logical link-layer channel capacity indicated by the maximum number of acknowledged packets that can be successfully transmitted from node *x* to *y* during timeslot *t*, respectively. The parameter $\theta_{x}^{m}\left(t\right)$ ($0\leq \theta_{x}^{m}\left(t\right)\leq 1$) determines a tradeoff between average queue backlogs and minimizing the objective function. As $\theta_{x}^{m}\left(t\right)$ gets closer to 0, routing is more controlled by backpressure routing than by the OF ranking. Node *x* selects neighbors with the minimum weight as the potential parents.

To adjust $\theta_{x}^{m}\left(t\right)$ adaptively according to the degree of congestion, BRPL uses the online algorithm QuickTheta. This algorithm computes $\theta_{x}^{m}\left(t\right)$ using the smoothed queue length and the parameter $\beta_{x}\left(t\right)$ which indicates the mobility condition. $\beta_{x}\left(t\right)$ also is adaptively adjusted by the online algorithm QuickBeta which observes the change of one-hop neighbor nodes. These adaptive algorithms make RPL smoothly switch to backpressure routing when traffic load is heavy or node mobility is high.

Performance was evaluated through Cooja simulations and real-testbed experiments using the FIT IoT-LAB testbed [48]. The authors used 100 M3 Open Nodes from the Grenoble site offered by the FIT IoT-LAB testbed, and constructed a network consisting of five roots and 95 sensor nodes. For the MAC layer, a CSMA/CA driver combining with MiCMAC was used. The radio duty cycling was disabled. BRPL was compared with the basic RPL and backpressure routing. The basic RPL and BRPL both use the same ETX OF with the same (default) settings.

In the experiments with traffic bursts, the packet loss rate of BRPL was over 4.5 times lower than that of RPL because BRPL can adapt to the traffic dynamics with the QuickTheta algorithm. In the experiments with the data sensing rate fixed over time, RPL showed the highest packet loss rate because RPL always chooses the optimal path based on the OF. Since BRPL and backpressure routing use suboptimal paths when the traffic load is high, they can enjoy lower packet loss rates and higher throughput than RPL. BRPL outperformed backpressure routing in terms of end-to-end delay when the traffic load was light. Since backpressure routing routes packets relying only on congestion gradients, it suffers from long end-to-end delays.

#### 4.2.2. Multipath Opportunistic RPL Algorithms

**Multipath opportunistic routing by sender’s decision.** Pavkovic et al. propose a multipath opportunistic RPL over IEEE 802.15.4 to support delay-sensitive alarm applications [32]. In this scheme, each packet is assigned a deadline according to the class of service, and each node has packets ordered by their deadlines in the queue. Whenever a packet is dequeued for transmission, the node tries to find a parent candidate to which the packet is correctly transmitted within the local time budget constraint. Since the authors consider the IEEE 802.14.5 in beacon-enabled mode, the transmission delay means the delay until the superframe of the candidate starts plus the average delay until the candidate correctly receives the packet. If more than one parent candidate can satisfy the delay constraint, the one with the least ETX is selected. As a consequence, a node opportunistically forwards packets over multiple parents on a per-packet basis. For efficient interoperability between multipath RPL and IEEE 802.15.4, they modified the cluster-tree formation mechanism of IEEE 802.15.4 so that a node can associate with several parent nodes by adequately organizing superframes at the MAC layer.

For performance evaluation, the authors compared the opportunistic RPL with the basic RPL using WSNet/Worldsens simulator in which they ported the Contiki RPL implementation. For the MAC layer, beacon-enabled IEEE 802.15.4 was used. The two RPL versions both took advantage of the 802.15.4 superframe scheduling in order to adaptively choose a parent. Simulation results show that the opportunistic RPL improves delay and packet delivery ratio for the traffic types with the delay constraints. Although the primary goal of this scheme is to satisfy the delay constraint rather than to reduce congestion, the simulation results show that the scheme can enjoy improved network lifetime and fairness since traffic is spread more uniformly over all feasible parents.

**ORPL.** ORPL [27], proposed by Duquennoy et al., is an opportunistic routing protocol that supports any-to-any, on-demand traffic as well as upward and downward traffic. In ORPL, a node anycasts a packet instead of choosing a next hop and then forwarding a packet to it. For upward routing, a next hop is selected during transmission. That is, the first neighbor that (1) wakes up, (2) successfully receives the packet, and (3) is the closer to the root, is supposed to acknowledge the packet and forward it. Therefore, forwarding decision is made at the receiver rather than at the sender. This is one of the major differences between ORPL and the opportunistic RPL proposed by Pavkovic et al [32]. For routing downwards, ORPL uses the notion of ‘routing set’. The routing set of a node, which is the set of nodes in the sub-DODAG rooted at the node, provides the information on whether a node is on a path to the destination or not. A node forwards a packet downwards if the node (1) wakes up, (2) successfully receives the packet, (3) is farther from the root, and (4) has the destination in its routing set. For any-to-any traffic, a packet is routed upwards to a common ancestor, and then downwards to the destination. ORPL aims to achieve space efficiency and scalability by using routing sets instead of routing tables and by adopting bitmaps or bloom filters to store routing sets.

As a routing metric, ORPL uses EDC (Estimated Duty Cycled wake-ups) [49] which reflects the expected number of duty cycled wakeups required to reach the root. EDC is an adaptation of ETX for energy-efficient anycast routing. The EDC of node *i* is computed recursively as follows.

(15) $$EDC\left(i\right)=\frac{1}{\sum_{j\in F_{i}}p_{ij}}+\frac{\sum_{j\in F_{i}}p_{ij}EDC\left(j\right)}{\sum_{j\in F_{i}}p_{ij}}$$

where $F_{i}$ is the set of node *i*‘s forwarders, $p_{ij}$ is the probability that node *i* successfully sends a packet to node *j*. The first term approximates the expected delay from node *i* to any forwarder, the second term the expected delay from a forwarder to the sink. The forwarder set $F_{i}$ consists of *i*‘s neighbor nodes *j* that provide routing progress, i.e., EDC(j)<EDC(i)−w, where *w* is a weight for the balance between opportunism and the number of hops.

The authors evaluate ORPL using the Indriya testbed [50] consisting of 135 telosB nodes. The ContikiMAC which they used has a phase-lock mechanism where senders record their neighbor’s wake-up phase, and use it to make the next transmissions cheaper. To avoid losing synchronization when long wakeup intervals are used, they extended the ContikiMAC default phase lock guard time from 15 to 63 ms. Evaluation results show that in a data collection scenario, ORPL outperforms the basic RPL, collection tree protocol (CTP) [51], opportunistic routing for WSN (ORW) [52], and low-power wireless bus (LWB) [53] in terms of packet delivery ratio, end-to-end delay, and duty cycle used as a metric for energy efficiency. For one-to-many and any-to-any traffic, ORPL outperforms RPL in terms of latency, PDR, and duty cycle.

However, the authors state that since duty-cycled anycast used by ORPL sometimes causes multiple nodes to forward the same packet and to generate duplicates, they filtered out duplicates at the routing layer to reduce unnecessary forwarding. Experimental results show that the duplicates accounted for 9% to nearly 50% of the total traffic. The authors point out that this is an inherent drawback of ORPL, which is exacerbated as the wakeup interval decreases. Also, Parasuram et al. point out that the routing set for downward routing cannot be used with the nonstoring mode of operation [42].

**ORPL-LB.** Motivated by the fact that ORPL cannot avoid energy hotspots without an explicit load balancing policy, Michel et al. propose ORPL-LB [33], a load balanced extension of ORPL. Like ORPL, ORPL-LB enables the receiver to make a forwarding decision. That is, the sender node transmits continuously until any of its neighbors wakes up, receives the packet, decides to forward it and acknowledges it. However, ORPL-LB considers a wake-up interval which affects a node’s availability as a forwarder in the future. Each node adjusts its wake-up interval continuously to reach the target duty cycle, which is pre-defined or determined at runtime. If the average duty cycle is lower than the target duty cycle, the node decreases the wake-up interval in order to reach the target. This causes lightly loaded nodes to wake up more frequently and to hear and forward more traffic. As a result, these nodes’ loads increase whereas their overloaded neighbors’ loads decrease. Conversely, a node with a high duty cycle increases its wake-up interval, which leads to reduction of traffic load and idle energy consumption.

The authors evaluated performance using a periodic data collection scenario in the Indriya testbed which had about 93 Tmote Sky nodes. For ORPL, they set the wakeup interval to 500 ms which was optimal for the experiment. Experimental results show that compared to ORPL, ORPL-LB reduces the highest duty cycle at the cost of increasing the average duty cycle slightly. Reducing the highest duty cycle means that energy consumption is better balanced between nodes. By making many nodes fail almost at the same time, ORPL-LB extends the network lifetime by 33% compared to ORPL.

**ORPL-DT.** Kang et al. observed that ORPL suffers from unreliable routes when downward traffic is dominant [34]. The reason is that since ORPL updates routing metrics using upward packets only, it fails to provide up-to-date EDC information if there is a lack of upward traffic. To enhance ORPL for diverse traffic patterns, the authors propose ORPL-DT (ORPL for diverse traffic) [34] which constructs reliable routes in diverse traffic scenarios by exploiting both upward and downward packets to update the EDC metric which ORPL uses.

EDC of a node represents its proximity to the root in an opportunistic routing environment and is defined as already shown in Equation (15). Note that $p_{ij}$ in Equation (15) is the packet reception ratio (PRR) when node *i* sends a packet to node *j*. The first term in Equation (15) is called the single hop EDC, which indicates the expected time to deliver a packet from the node to any of its forwarders. The authors are focused on how to measure the single hop EDC. Although the definition of the single hop EDC requires that each node collect PRR for all of its potential upward forwarders, ORPL practically updates its single hop EDC simply by measuring latency from transmitting an upward anycast packet to receiving an ACK from any upward forwarder, without measuring individual PRR values. However, each node cannot update EDC in the same way by exploiting downward transmission. The reason is that EDC represents the opportunistic route quality between the node and the sink, whereas the downward transmission results represent the route quality between the node and its subtree nodes.

To address the problem, in ORPL-DT, each node maintains both its EDC metric and PRR for each potential upward forwarder. For each upward transmission, a node calculates PRR by measuring the number of retransmissions until receiving an ACK from the upward forwarder. Using the updated PRR, the node calculates the current single hop EDC. Then, using the updated single hop EDC, it updates PRR for each of the other candidate upward forwarders. For each downward transmission, each node enables the downward forwarder receiving the packet to update the PRR information by piggybacking the number of retransmissions in the downward packet. Since the node sending the downward packet is a potential upward forwarder of the receiver, the receiver can update the single-hop EDC metric for its upward forwarder.

Performance of ORPL-DT was evaluated through experiments on a testbed consisting of a sink node and 30 telosB motes with the ContikiOS. They used ContikiMAC with the default wakeup interval of 500 ms. They compared ORPL-DT and ORPL for diverse scenarios varying the interpacket intervals of downward and upward traffic. The results show that in all scenarios, ORPL-DT outperforms ORPL in terms of downlink PRR, uplink PRR, duty cycle, and routing control overhead.

However, for a severely asymmetric link, the PRR information updated via downward transmissions may not accurately reflect PRR for the potential upward forwarder.

#### 4.2.3. LB-RPL

Liu et al. observed that uneven deployment of sensor nodes in large areas and heterogeneous traffic patterns in a network may cause workload imbalance which shortens network lifetime. They propose LB-RPL [35], which detects workload imbalance and optimizes the data forwarding path by considering both workload distribution and link-layer communication qualities. To alleviate workload imbalance, LB-RPL adopts two approaches. First, LB-RPL forwards data using multiple parent nodes. Each node considers top *k* parent nodes as potential next-hop nodes for data forwarding. The probability $f_{ij}$ of node *i* forwarding a data packet to parent node *j* is calculated based on the packet drop probability $p_{ij}^{c}$ between the two nodes due to the channel condition.

(16) $$f_{ij}=\frac{\left(1-p_{ij}^{c}\right)}{\sum_{j=1}^{k}\left(1-p_{ij}^{c}\right)}$$

Second, LB-RPL sets a timer for DIO message transmission in proportion to workload measured using a buffer utilization counter. Depending on the application and the underlying hardware, a buffer utilization counter is defined as the average number of packets in the queue over a certain time period or the total number of packets queued in the buffer. If a parent node has heavy workload in the current round, it will add more delay for DIO message transmission in the next round. Since the long delay lowers the probability that a parent with heavy workload is in top *k* list in its children’s parent table, workload imbalance can be mitigated.

Performance was evaluated via ns-2 simulation using IEEE 802.15.4 at the PHY and MAC layer. In the simulation, LB-RPL successfully spread out the workload among the nodes around the data collector and achieved a higher packet delivery ratio and a shorter average delay than RPL for all buffer sizes.

However, Kim et al. point out that DIO packet reception time may not exactly reflect the queue utilization due to DIO packet losses and that LB-PRL may cause slow recovery and the herding effect [33].

#### 4.2.4. RPL using the Heuristic Load Distribution Algorithm

Moghadam et al. propose a multipath routing mechanism based on RPL and a load distribution mechanism to equalize traffic load between nodes of the same level in a DODAG using the heuristic load distribution algorithm (HeLD) [36]. To construct a multipath DODAG, a node *i* maintains a parent set $P_{i}$ using DIO messages received from more than one neighbor node with the minimum hop count. Node *i* computes its rank *R*(*i*) as follows.

(17) $$C_{ij}=R\left(j\right)+c_{ij}$$

(18) $$w_{j}\left(i\right)=1-\frac{C_{ij}}{\sum_{j\in P_{i}}C_{ij}}$$

(19) $$R\left(i\right)=\underset{j\in P_{i}}{\sum }w_{j}\left(i\right)C_{ij}$$

where $c_{ij}$ denotes the cost of link (*i, j*), $C_{ij}$ the route cost from node *i* to the sink through node *j*, $w_{j}\left(i\right)$ parent *j*’s share of node *i*’s output traffic rate. The share $w_{j}\left(i\right)$ is initialized as shown in Equation (18), but adaptively updated by their load distribution algorithm. The authors used ETX as the main routing metric (i.e., $c_{ij}$). Since the rank must increase monotonically, any parent with a greater rank is removed from the parent set and the rank is recomputed.

The authors formulate the cost-efficient load distribution optimization problem as a network flow problem. To approximately solve the problem using local information only, they propose the heuristic load distribution (HeLD) algorithm. In HeLD, each node compares the input traffic rates of its parents in a pairwise manner. If the input traffic rates are not close enough between two parents, the traffic shares are gradually adjusted to be equalized between the parents, i.e., the nodes of an equal depth from the sink. In this way, HeLD tries to equalize energy consumption rates between the nodes of an equal depth from the sink while minimizing the total transmission cost. To this end, each node must obtain its parent nodes’ incoming traffic rates through feedback messages such as ‘hello’ or ‘ACK’.

For performance evaluation, the authors compared this scheme to the standard single-path RPL and a node-disjoint multipath routing algorithm via simulation using OMNET++ and MATLAB. At the data link layer, unslotted CSMA-CA over IEEE802.15.4 was used. Simulation results show that this scheme enhanced the network lifetime and throughput compared to the other schemes, but caused more link drops due to utilization of less reliable links.

#### 4.2.5. Energy-Aware Load Balancing

**Energy-balancing based on energy bottlenecks.** Iova et al. propose a multipath RPL scheme to balance energy consumption [37]. This scheme aims at maximizing the lifetime of the most constrained node named bottleneck rather than simply using the nodes with large residual energy to forward traffic. To this end, each node identifies the energy bottleneck nodes along the paths from itself to the root and splits its traffic among all its parents so that the bottlenecks can be equally charged.

This scheme uses a metric called ELT (expected life time) which is the time until a node runs out of energy if it keeps on forwarding the same amount of traffic. The ELT of a node N is calculated as follows.

(20) $$ELT\left(N\right)=\frac{E_{res}\left(N\right)}{\sum_{P\in Parent\left(N\right)}\frac{\alpha_{P}\times T_{tot}\left(N\right)\times ETX\left(N,P\right)}{DATA\_RATE}\times P_{TX}\left(N\right)}$$

where $\underset{P\in Parent\left(N\right)}{\sum }\alpha_{P}=1$. $E_{res}\left(N\right)$, $T_{tot}\left(N\right)$, $P_{TX}\left(N\right)$, $\alpha_{P}$, *ETX(N,P)*, and *DATA_RATE* are the residual energy, the traffic node *N* has to forward, the transmission power of node *N*, the portion of traffic forwarded to parent *P*, ETX of the link between *N* and *P*, and the data rate, respectively. *DATA_RATE* is assumed to be the same for all the nodes in the network. $\frac{P_{TX}\left(N\right)}{DATA\_RATE}$ indicates the energy drained per transmitted bit. Since the rank of nodes must monotonically increase downwards in a DODAG, ELT cannot be used to compute the rank. The rank of a node is calculated using its preferred parent’s rank and a constant step value.

The bottleneck of a path can be defined as a node which has the minimum ELT along the path. Several parents may lead to the same bottleneck. Each node maintains a list of all its bottleneck nodes and estimates the ELT of each bottleneck. To propagate the last updates on bottlenecks in the network, a DIO message contains a list of bottlenecks with the information for each of them: the bottleneck ID, the ratio of the traffic forwarded by this node to the bottleneck, the existing traffic forwarded by the bottleneck to the sink, the residual energy, and the spent energy to transmit one bit at the bottleneck. With this information, a node *N* estimates the effect of its traffic on the lifetime of bottleneck *B* by adding the ratio of its own traffic to the traffic of *B*.

To select a preferred parent, each node calculates the lifetime of the bottlenecks assuming that it sends all its traffic to one parent, and selects a node that maximizes the lifetime of the bottleneck. After choosing a preferred parent, the node removes from the parent set all the nodes that have larger rank than its rank. Each node splits traffic among all its parents in proportion to ELT of the corresponding bottleneck node.

For performance evaluation, they simulated RPL using WSNet. At the MAC layer, the IEEE 802.15.4 with the beacon-enabled mode was used. The authors compared their multipath RPL scheme with single-path RPL versions which use different routing metrics. Simulation results show that their multipath scheme achieved a better network lifetime than the other (single-path) RPL schemes and better stability than the standard RPL in that their scheme changed the parent less frequently.

However, since estimation of bottlenecks’ ELT requires that each node obtain the information on its bottlenecks and calculate the ratio of traffic forwarded to the bottleneck recursively, the scalability problem due to computation and communication overhead needs to be investigated. Parasuram et al. point out the increased risk of L2 fragmentation [42]. Since the sizes of DIO messages increase to carry the information about bottlenecks, DIO messages may be fragmented into multiple fragments each of which may take a different path and be lost.

**Cross-layer approach.** Nassiri et al. adopt a cross-layer approach to energy-aware load-balanced routing [38]. Since this work uses the beacon-enabled IEEE 802.15.4 in which beacon frames are sent more frequently than DIO messages, beacon frames are used to inform child nodes of the metrics such as the upstream load estimate and the upstream energy estimate. The work is based on a modified cluster-tree MAC, which was proposed by Pavkovic et al. [32] and permits selecting multiple parents. While constructing a DODAG, each node builds a list of candidate parents by screening out nodes having low RSSI or low upstream energy. If the queue occupancy exceeds a predefined threshold, the probability of parent *p* being selected as the preferred parent for the current packet is calculated as follows.

(21) $$\mathrm{Pr}\left(p\right)=1-\frac{\alpha_{1}\times load\left(p\right)}{\sum_{q\in tPars}load\left(q\right)}-\frac{\alpha_{2}\times dist\left(p\right)}{\sum_{q\in tPars}dist\left(q\right)}$$

where *tPars* is the set of this node’s parents, *load*(*p*) the upstream load estimate announced by *p*, and *dist*(*p*) the remaining time to the start of *p*’s superframe. Positive coefficients *α*_1_ < *α*_2_ are set so that a higher priority is given to a parent with early superframe start time. If the number of packets in the sender’s transmission queue remains below a threshold, *α*_2_ is set to 0 so that a parent with a smaller upstream load estimate relays more traffic.

Performance was evaluated via WSNet simulation using 144 nodes. At the MAC layer, the beacon-enabled IEEE 802.15.4 was used. The authors compared their scheme to the standard RPL varying the offered load. They evaluated two versions of their work—one using both the upstream load estimate and the superframe distance, the other one using the superframe distance only. In comparison with the standard RPL, both versions achieved a higher PDR and extended the network lifetime for low and moderate traffic load. In terms of delay, the version using only the superframe distance outperformed the standard RPL. The version using both metrics experienced a longer delay than the standard RPL for high traffic load because it selects less congested but longer paths.

#### 4.2.6. M-RPL

Lodhi et al. proposed M-RPL, a multipath extension of RPL which supports temporary multipath routing only while congestion occurs over a path [22]. Each node detects congestion by monitoring packet delivery ratio (PDR) on receiving each packet. To calculate PDR, each node must have the information of the expected data rate of each child node. Therefore, each node uses DAO messages to send its parent node the information of the current forwarding rate. If the average PDR during a congestion interval (CI) decreases below a predefined threshold, the node is deemed congested.

Once a node detects congestion, it sends a congestion notification (CN) to its child nodes using a periodic DIO message. A node receiving a DIO message with CN starts forwarding packets using both the original parent and any other node chosen from the parent table. To avoid forwarding packets to congested nodes, each node saves the CNs of its neighbors in the parent table. If a child node does not hear CN from its parent any more, it stops splitting traffic.

For performance evaluation, the authors compared M-RPL to the basic RPL (with MRHOF) over the IEEE 802.15.4 MAC via Cooja simulation. M-RPL significantly outperformed RPL in terms of average throughput particularly when the data rates were high. M-RPL also showed slightly lower per-bit energy consumption than RPL. However, M-RPL did not reduce the end-to-end delay significantly because the delay of M-RPL increased while constructing multiple paths on detecting congestion. As congestion became persistent, the end-to-end delay stabilized for both protocols and M-RPL showed a shorter delay than RPL.

#### 4.2.7. Bird Flocking Congestion Control Algorithm

A flock-based congestion control approach for wireless sensor networks was proposed by Antoniou et al [54]. The approach is based on the idea of guiding packets towards a global attractor (the sink in WSNs), while trying to avoid obstacles (failing nodes and congested regions). The movement direction of a packet group is determined using repulsion and attraction forces between packets, the field of view and the artificial magnetic field in the direction of the artificial magnetic pole (sink). The field of view (FoV) which limits the range of potential next hops is used to keep packets from being trapped in loops and to prevent the same node from always being considered.

In the bird flocking model, each packet is supposed to be repelled from neighboring packets located on nodes one hop away. The repulsion force is proportional to the number of packets on nodes one hop away, which is obtained using broadcast control packets. In contrast, each packet is attracted to neighboring packets located on nodes two hops away and the attraction force is proportional to the number of packets on nodes two hops away. Since nodes two hops away are outside the transmission range, the number of packets on nodes two hops away is inferred by measuring the number of packets transmitted successfully from nodes one hop away to nodes two hops away. The repulsion and attraction forces are synthesized using the desirability function. A node within the FoV with the highest desirability function value is selected as a next hop. In this way, packets are ‘flying’ through the network while being attracted to nodes with low wireless channel loading, and being repelled from nodes with high buffer occupancy.

Hellaoui et al. apply a similar bird flocking model to control congestion in CoAP/RPL/6LoWPAN networks [39]. In their approach, the ZoR (zone of repulsion) of a node is the set of its parents and children and the ZoA (zone of attraction) the set of direct ascendants of its parents and direct descendants of its children. A packet tries to avoid more congested nodes in ZoR and to follow less congested nodes in ZoA. The congestion level of a node is defined as the buffer filling ratio. To estimate the buffer filling ratios of other nodes accurately without incurring high overhead, the authors propose eavesdropping data messages instead of using control messages. For each packet, the next hop is selected satisfying two conditions: (1) the next hop should be the least congested in ZoR, (2) the successors of the next hop should be the least congested in ZoA.

Through Cooja simulation, performance of the proposed scheme was evaluated. However, the authors only showed the effect of tuning the retransmission-related parameters, but did not clarify how effective the bird flocking approach to next hop selection in RPL was in mitigating congestion compared to the existing RPL approaches.

#### 4.2.8. CA-RPL

Tang et al. propose CA-RPL which is a multipath routing scheme for congestion avoidance. CA-RPL is focused on performance improvement in emergency scenarios where monitoring information about sudden events needs to be quickly and reliably transmitted to the sink [40]. To this end, the authors propose a new metric, ‘minimized delay metric’ (or DELAY_ROOT) which is defined as the cumulative sum of the hop-by-hop forwarding delays along the path from a node to the DODAG root. The minimized delay metric is calculated based on the ContikiMAC duty cycling mechanism. To compute the weight of a path, each node uses a composite routing metric of ETX, its rank, the number of received packets in a time period, and DELAY_ROOT. After ranking parents according to their weights, a node selects two parent candidates which have the highest weights. Packets are distributed over the parents in proportion to the path weight.

The authors evaluated performance via Cooja simulation with the ContikiMAC duty cycling protocol. CA-RPL outperformed the basic RPL in terms of packet reception number (PRN) of the root node per unit time, overall throughput of the network, packet loss rate, and average latency.

However, since the amount of metric information to be carried in a DIO message is not small, control overhead needs to be analyzed. In particular, as Parasuram et al. point out, adding a lot of additional fields to the DIO message increases a risk of L2 fragmentation [42].

#### 4.2.9. H-RPL

Guo et al. propose a resource aware hierarchical RPL(H-RPL) for heterogeneous wireless IoT networks consisting of nodes with different resources and capabilities [41]. To support multiple tasks, H-RPL can use different routing metrics and OFs at different tiers of an H-DODAG topology. H-RPL also enables each node to determine its routing type, such as upward routing, nonstoring downward routing, or storing downward routing based on the availability and requirement of resources.

To indicate the routing type of a node, H-RPL uses a modified MOP (mode of operation) mechanism. In the original RPL, all nodes that join a DODAG as routers use the same MOP as the root, and nodes with different MOPs join only as leaves. In contrast, H-RPL allows mixed MOPs in a DODAG. Also, H-RPL allows the MOP of a node to be adaptively changed depending on available resources (e.g., expected routing lifetime), required resources (e.g., required routing memory), and neighbor nodes’ MOPs. Since a higher MOP requires more memory to support more routing functions, H-RPL shifts routing workload from nodes with lower MOPs to nodes with higher MOPs.

To avoid congestion by upward data packets, H-RPL distributes upward packets among parent nodes based on the queue occupancy index (QOI) which is defined as the average queue utilization. A node ranks its parents according to their QOIs. The higher the QOI, the lower the ranking. Upward packets are distributed between parents in proportion to the ranking.

Performance of H-RPL was compared to that of the standard RPL using NS-2 simulator with IEEE 802.15.4 MAC and PHY. Nonstoring nodes were battery-powered and able to buffer five packets, whereas storing nodes were mains-powered and able to buffer ten packets. Since the simulated network consisted of storing nodes and nonstoring nodes, H-RPL operated in the mixed MOP = 1 and 2, and the standard RPL operated in MOP = 1. Simulation results show that H-RPL outperformed the standard RPL in terms of upward data PDR, downward data PDR, and network lifetime.

### 4.3. Congestion Control for DAO Packets

While most studies pay attention to congestion due to data traffic, Tripathi et al. focused on how to control congestion due to destination advertisement object (DAO) packets [26]. DAO packets are generated when the destination information needs to be propagated upward along the DODAG. For instance, a global repair may cause every node to generate DAO packets toward the root. Their study was motivated by the observation that for a very large network, RPL nonstoring mode operation requires a large buffer space. This is counter-intuitive because in the nonstoring mode, a node does not have to store routing table information. The authors found that such a large buffer is needed because each node needs to store DAO packets from its sub-DAG. If buffer space is not available, important data packets as well as DAO packets may be lost.

The authors propose algorithms to control DAO emissions by using an adaptive DelayDAO timer at each node instead of the fixed timer in the standard RPL. For the nonstoring mode, they try to distribute the generation time for DAO packets within a time interval to prevent many DAO packets from propagating at the same time. Based on the assumption that the number of nodes tends to increase exponentially with the rank, the DelayDAO timer value $T_{DD}$ is randomly chosen in the interval that increases exponentially with the rank as follows:

(22) $$T_{DD}=random\left(K*Base^{Rank-1},\;K*Base^{Rank}\right)$$

where *K* and *Base* the adaptive parameters. To minimize buffer occupancy in nodes near the DAG root, the parameters *K* and *Base* are tuned by a distributed algorithm or a centralized algorithm. In the distributed algorithm, each node tunes *Base* and *K* by speculating on possible congestion based on RTT measured using DAO packets. If the DAG roots have much more power and memory space than the other nodes in the network, the DAG roots can compute *Base* and *K* using the centralized algorithm and then distribute the computed values in the network.

For the storing mode, the authors consider the mechanism for DAO aggregation. If nodes in the storing mode aggregate DAOs from their subtree, the DelayDAO timer value is set inversely proportional to the rank because nodes of farthest distance from the DAG root need to release their DAO first.

Performance was evaluated using OMNET++ with 802.15.4 MAC/PHY model. They simulated a topology with 2442 nodes deployed in a city and random topologies with 200, 500, and 1000 nodes. To compute maximum required buffer space, no buffer drop was simulated. Simulation results show that the proposed algorithms outperform the basic RPL mechanism in terms of DAO reach time (i.e., the time required for a DAO of a node to reach the DAG root after the global repair or new DTSN is issued), maximum RAM consumption, data packet delivery latency, etc.

## 5. Discussion

In this section, we suggest future research challenges.

**Efforts to reduce resource consumption.** In designing protocols for LLNs, energy consumption is one of the major considerations. Most schemes reviewed aim to conserve energy directly or indirectly. Limited memory capacity also has been a critical constraint. Showing the memory requirements in RPL implementations of Contiki OS and TinyOS, Iova et al. point out that RPL has too large a memory footprint for resource-constrained devices [6].

However, the recent emergence of more powerful sensor motes indicates that the resource constraints in LLNs are changing. Developing Hamilton, an open source, 32-bit SoC-based mote, Kim et al. suggested new directions for the system architecture for networked sensors in the post-SoC era [55]. The authors also present the paradigm shifts to be considered in designing low-power network protocols. Among them, the issues closely related to RPL design are as follows. First, it has been pointed out that since protocol standards are not completely implemented due to resource constraints, devices using different implementations may not interoperate. As low-power 32-bit SoCs are becoming more capable and have more memory space, it may be feasible to implement the whole RPL standard, which will lead to better interoperability between devices. Second, the advanced radio features give new research opportunities such as adaptive data rate control, interference avoidance, and multiantenna diversity. Also, it should be considered that post-SoC motes have a different pattern of power consumption from the traditional motes in the sense that both the MCU and the radio become comparable power consumers in post SoC motes.

**Investigation via real implementation and experiments.** Kim et al. highlight the importance of evaluation through experiments using implementations on real embedded devices throughout their survey [7]. They point out that more than half of the research publications reviewed evaluated their proposals through simulation only, rather than with experiments on real testbeds. Our survey shows a statistic supporting their argument. Out of 23 publications reviewed in Section 4, only seven schemes evaluated performance using experiments on testbeds.

**Considerations on multiple RPL instances.** RPL allows an LLN to have multiple RPL instances with different QoS requirements. Each instance may have a different OF which uses different routing metrics and constraints. Kim et al. state that the statistics on the research papers from 2010 to 2016 indicate a lack of studies on multiple instances [7]. To the best of our knowledge, there are still a handful of studies on congestion control in an environment with multiple RPL instances.

Most of the efforts to apply multiple instances have been focused on smart grid (SG) environments where applications are heterogeneous in terms of QoS requirements. RFC 8036 on applicability of RPL in AMI (advanced metering infrastructure) networks states that multiple RPL instances can be supported in multiservice networks where different applications may require the use of different routing metrics and constraints, e.g., a network carrying both smart metering data (SMD) and distribution automation (DA) traffic [56]. Rajalingham et al. proposed an RPL extension, RPL-M, for a smart grid neighbor area network (SG NAN) which is expected to carry a mix of both periodic and critical traffic [57]. To differentiate QoS requirements at the network layer, RPL-M exploits multiple instances which use a hop count for periodic traffic and ETX for critical traffic. Pointing out that performance of RPL-M was evaluated in 802.11b networks, Banh et al. showed that multiple RPL instances can improve PDR and latency of each type of traffic in 802.15.4 networks [58]. More recently, Nassar et al. proposed an objective function, OFQS, with a tunable multiobjective metric, mOFQS, that is a composite metric of ETX, the delay between two nodes, and the remaining energy [59]. mOFQS adapts to the number of instances in the network depending on their criticality level by tuning its parameters accordingly. Performance of OFQS was evaluated using 67 client (M3) nodes and one server on FIT IoT-LAB testbed. OFQS was compared to RPL with MRHOF/ETX for critical traffic and OF0/HC for periodic traffic. Experimental results show that OFQS achieves better end-to-end delay, PDF, network lifetime, and load balancing.

Although using multiple RPL instances has not been the most active research topic, it is worth further investigating how to exploit multiple instances in order to meet different QoS requirements of heterogeneous applications while achieving load balancing. In [7], the authors state that to use multiple instances, one needs to consider implementation complexity and resource constraints of embedded nodes, but that it should be changed with modern devices that have more memory. We expect that the aforementioned emergence of powerful embedded nodes will be able to make multiple instances more viable.

**Relationship between TCP and RPL.** In the traditional WSN literature, it was widely accepted that the transport layer protocol must consume resources minimally; therefore, TCP was not considered as a good candidate. However, as IPv6-based LLNs have become prevalent, TCP has been spotlighted. According to the TCP usage guideline in IoT [60], one of the main reasons why TCP is considered for use in constrained node networks is that UDP-based communications may be limited or blocked in existing infrastructures. TCP designed for LLNs has been very limited in several aspects. For instance, BLIP TCP of TinyOS allows sender-side buffering for retransmission, but does not provide receiver-side buffering and sets the receive window to one MSS. Kim et al. investigated mutual influence between TCP and RPL through empirical tests on an IPv6-based LLN testbed [61]. For LLN endpoints, they used BLIP TCP and TinyRPL, which selects a parent based on uplink ETX. They found that TCP performance may degrade if RPL does not consider traffic load balancing.

As powerful low-power embedded devices with more processing power and larger memory space have become readily available [55], Kumar et al. claim that commodity LLN hardware has crossed a critical resource threshold to run full-scale TCP [62]. To show that a full-scale TCP can fit well within CPU and memory constraints of modern WSN platforms, they implemented a full-scale TCP, called TCPlp [62], leveraging the full-featured TCP in FreeBSD OS. According to their findings, another factor that makes a full-scale TCP viable is that in an LLN, a small window size is sufficient to fill the bandwidth-delay product and to achieve good TCP performance. They also showed that TCPlp achieves high throughput while consuming power comparably to CoAP which is an LLN-specialized protocol. The emergence of a full-scale TCP for LLNs calls for extensive studies on interaction between RPL and TCP. For instance, interoperability of multipath RPL schemes with TCP will likely be an interesting research topic, because out-of-order packets often have a negative impact on TCP goodput due to the fast retransmit mechanism.

**Cross-layer based efforts.** In [7], the authors argue that cross layer interoperability and/or performance optimization are not optional but mandatory to apply RPL in reality, given that RPL is designed for an LLN which is often a standalone network. As discussed in Section 3, several RPL schemes adopt cross-layer based approaches to congestion control and load balancing. Since the RPL schemes are focused on how to improve congestion control in a hop-by-hop manner, most of them interoperate with the lower layers. However, RPL should also interoperate with the transport layer to enhance end-to-end congestion control. For instance, since TCP for multihop wireless networks needs to deal with packet losses differently according to the cause of losses such as congestion, channel noise, and mobility [63], the cross-layer design issue involving TCP and RPL needs to be explored.

## 6. Conclusions

In this survey, we reviewed the research literature on congestion control and load balancing in RPL. To understand the trends, we categorized the proposals based on several criteria including target traffic patterns, routing metrics, cross-layer design approaches, and whether to exploit path diversity. In particular, the trends regarding routing metrics and cross-layer design approaches were summarized in more detail. It is notable that many schemes actively exploit path diversity by forwarding packets via multiple parents on a per-packet basis.

We also proposed future research directions which include RPL design considering more capable hardware platforms, investigation through real implementation and experiments, application of multiple RPL instances, interoperability with TCP, and cross-layer based design. We expect that the paradigm shifts mentioned in [55] will motivate researchers to revisit more issues. As diverse IoT application scenarios evolve, congestion control mechanisms for RPL are expected to continue to adapt to new requirements as well.

## Figures and Tables

**Table 1:** Congestion detection metrics.

Schemes	Congestion Detection Metrics
GTCC [16]	Packet generation rate subtracted by packet service rate
QU-RPL [17]	Queue utilization
Ullah et al. [18]	Queue utilization
PC-RPL [19]	Packet losses
OHCA [20]	Ratio of packet interarrival time to packet service time
CoAR [21]	Queue utilization, ratio of incoming traffic rate to outgoing traffic rate
M-RPL [22]	Packet delivery ratio

**Table 2:** Component metrics for routing and load distribution.

		HC	ETX	EDC	QU	Link delay	LQI	RSSI	Loss Rate	Traffic Rate	RE	Additional Metrics
GTCC	[16]						√			√		
QU-RPL	[17]	√	√		√							
Ullah et al.	[18]	√	√		√						√	
PC-RPL	[19]	√	√					√	√			subtree size
CA-OF	[28]		√		√							
OHCA	[20]		√		√	√						
CoAR	[21]		√		√						√	no. of potential children/no. of neighbor nodes
Marco et al.	[29]						√			√		busy channel prob., reliability constraint
Farooq et al.	[30]	√	√		√	√						available bandwidth
BRPL	[31]		√		√							logical link layer channel capacity, change of no. of one-hop neighbors
Pavkovic et al.	[32]	√	√			√						deadline constraints
ORPL	[27]			√								
ORPL-LB	[33]			√								duty cycle
ORPL-DT	[34]			√								
LB-RPL	[35]				√				√			
Moghadam et al.	[36]	√	√							√		
Iova et al.	[37]		√							√	√	
Nassiri	[38]				√			√		√	√	superframe distance
M-RPL	[22]											PDR
Hellaoui et al.	[39]				√							
CA-RPL	[40]		√			√				√		wake-up interval
H-RPL	[41]				√						√	available/required memory

Acronyms: HC (hop count), EDC (estimated duty cycled wake-ups), QU (queue utilization), RE (residual energy).

**Table 3:** Cross-layer design schemes.

	Direction of Information Flow	Relevant Metrics and Parameters	How the Layered Architecture is Violated	Objectives
GTCC [16]	Upward	LQI	Calculating the rank considering LQI	To detect congestion and node failure and unavailability
Ullah et al. [18], CoAR [21]	Upward	Residual energy	Parent selection considering remaining energy	To avoid selecting energy-constrained nodes
PC-RPL [19]	Back and forth	RSSI	Jointly controlling transmission power and routing topology	To address the hidden terminal problem
Marco et al. [29]	Upward, Downward	LQI, Busy channel probability, Max. no. of backoffs and retransmissions at the MAC layer, Reliability constraint	Calculating link reliability and traffic load distribution considering bad channel probability (LQI), busy channel probability, MAC parameters, and power consumption, reliability constraint given by the application	To extend reliability metric by considering packet losses due to MAC contention To distribute load while keeping the minimum reliability required by the application
Pavkovic et al. [32]	Downward	Deadline constraints	Selecting a node that satisfies the deadline constraint which is assigned on packet generation	To meet the deadline that is assigned to each packet
ORPL-LB [33]	Back and forth	Wake-up interval	Adapting the wake-up interval by comparing the current duty cycle and the target duty cycle	To balance load by adjusting nodes’ wake-up intervals
Iova et al. [37]	Upward	Residual energy	Identifying the bottleneck nodes in terms of Expected Lifetime metric (=time until a node will run out of energy)	To detect energy bottleneck nodes and to spread the traffic among them
Nassiri [38]	Upward	RSSI, Residual energy, Superframe distance,	Using RSSI for the initial DODAG construction and considering the upstream energy estimate and superframe distance for periodic parent selection	To prevent selecting parents with low quality or low energy and to reduce end-to-end delay in the beacon-enabled mode
H-RPL [41]	Upward	Residual energy, Available/required memory	Determining the routing type adaptively considering the critical resource status such as residual energy and available/required memory	To balance the routing workload between nodes considering the resource status and requirement

**Table 4:** Protocols forwarding packets via a single parent.

	Main Feature	Routing Metrics & Constraints	Evaluation Tool	Evaluation Metrics
GTCC [16]	Designing the parent change procedure using a potential game. Detecting congestion based on the difference of packet transmission rate and the packet service rate. Notifying congestion explicitly by sending a DIO packet with the CN bit set.	Link quality indicator (LQI), transmission rate	Cooja simulation	Packet losses, throughput, average hop count
QU-RPL [17]	Selecting a parent based on queue utilization, hop count, and ETX	ETX, queue utilization, hop count	Real testbed experiment	PDR, routing overhead, scalability
Ullah et al. [18]	Selecting a parent based on queue utilization, residual energy, and ETX	ETX, queue utilization, residual energy	Cooja simulation	PDR, power consumption
PC-RPL [19]	Addressing the hidden terminal problem and load imbalance by jointly controlling transmission power and routing topology	Hop count, ETX, queue losses, link losses, RSSI	Real testbed experiment	PRR, hop count, end-to-end ETX, parent change frequency, routing overhead, total transmission count
CA-OF [28]	Using a weighted sum of ETX and buffer occupancy	ETX, buffer occupancy	Cooja simulation	PDR, number of lost packets, throughput, energy consumption
OHCA [20]	Adopting both resource control (parent change if available) and traffic control (sending rate adaptation). Detecting congestion by monitoring if the arrival rate exceeds the service rate	Buffer occupancy, ETX, queuing delay	Cooja simulation	Throughput, weighed fairness index, end-to-end delay, energy consumption, number of lost packets
CoAR [21]	Using TOPSIS to select the best alternative parent. Adjusting the threshold for congestion detection according to the buffer occupancy and the incoming/outgoing traffic	ETX, queue utilization, residual energy, neighborhood index	Cooja simulation	PRR, end-to-end delay, packet loss rate, throughput, power consumption
Marco et al. [29,46]	Proposing two metrics exploiting the information from the MAC layer: R-metric representing end-to-end reliability and Q-metric representing the optimal traffic for balanced energy consumption	LQI, busy channel probability, traffic rate	Testbed experiment (using TelosB platforms)	End-to-end node reliability, average node power consumption
Farooq et al. [30]	Extending the original RPL for a wireless sensor network with multiple sinks	Hop count and tie-breaking metrics (available bandwidth, delay, MAC layer queue occupancy, ETX)	Cooja simulation	PDR, delay, total retransmissions

**Table 5:** Protocols forwarding packets via multiple parents.

	Main Feature	Routing Metrics & Constraints	Evaluation Tool	Evaluation Metrics
BRPL [31]	Switching smoothly and adaptively between the RPL OF with backpressure routing. Considering mobility.	Queue backlogs, ETX	Cooja simulation, FIT IoT-LAB testbed	Packet loss, end-to-end delay, communication overhead
Pavkovic et al. [32]	Opportunistically forwarding packets over multiple parents satisfying the delay constraint on a per-packet basis	Delay to the sink, deadline constraints, ETX	WSNet/Worldsens	PDR, incurred delay, overhead
ORPL [27]	Opportunistic routing protocol supporting upward, downward, and any-to-any on-demand traffic	EDC (Estimated Duty Cycled wake-ups)	Indriya testbed experiment	PDR, latency, duty cycle
ORPL-LB [33]	Opportunistic forwarding. Adjusting the wake-up interval depending on the traffic load	EDC, average duty cycle	Indriya testbed experiment	PDR, latency, duty cycle,
ORPL-DT [34]	Opportunistic forwarding. Improving the reliability of routes by updating the routing metric using both upward and downward traffic	EDC	Real testbed experiment	Uplink/downlink packet reception ratio (PRR), PRR fairness, no. of routing messages
LB-RPL [35]	Forwarding data using multiple parent nodes. Setting the DIO message transmission timer in proportion to workload	Packet drop probability, buffer utilization counter	Ns-2 simulation	Packet delivery ratio, packet loss, packet delivery delay
Moghadam et al. [36]	Equalizing traffic load share between parents of an equal depth from the sink while minimizing transmission cost	Hop count, ETX	Simulation using MATLAB, OMNET++	Network life time, throughput, collisions, link drops
Iova et al. [37]	Splitting traffic between multiple parents to spread traffic load uniformly among energy bottleneck nodes	ELT (Expected Lifetime)	WSNet	PDR, energy consumption, network lifetime, no. of parent changes
Nassiri [38]	Adopting a cross-layer approach using RSSI and superframe distance.	RSSI, superframe distance, remaining energy, load	WSNet	PDR, Delay, Lifetime
M-RPL [22]	Initially establishing a single path but providing temporary multipath routing during period of congestion. Detecting congestion relying on average PDR		Cooja simulation	Throughput, end-to-end delay, energy consumption
Hellaoui et al. [39]	Using a bird flocking model	Buffer filling ratio	Cooja simulation	Latency, Packet loss ratio
CA-RPL [40]	Using a delay metric calculated considering the duty cycle at the MAC layer. Splitting traffic over two best parents	ETX, minimized delay to the DODAG root, no. of received packets	Cooja simulation	Throughput, packet loss ratio, latency
H-RPL [41]	Allowing heterogeneous, mixed, adaptive routing types based on available/required resource. Shifting routing workload from nodes with less resource to nodes with more resources. Load balancing based on queue utilization.	Available/memory, required memory, expected routing lifetime	Ns-2 simulation	PDR, energy consumption, network lifetime
